# Topical Antinociceptive Effect of *Vanillosmopsis arborea* Baker on Acute Corneal Pain in Mice

**DOI:** 10.1155/2014/708636

**Published:** 2014-02-10

**Authors:** Laura Hévila Inocêncio Leite, Gerlânia de Oliveira Leite, Thales Silva Coutinho, Severino Denício Gonçalves de Sousa, Renata Souza Sampaio, José Galberto Martins da Costa, Irwin Rose Alencar de Menezes, Adriana Rolim Campos

**Affiliations:** ^1^Programa de Pós-Graduação em Bioprospecção Molecular, Universidade Regional do Cariri, 63105-000 Crato, CE, Brazil; ^2^Universidade de Fortaleza, Avenida Washington Soares 1321, 60811-905 Fortaleza, CE, Brazil

## Abstract

This study aimed to assess the possible topical antinociceptive activity of *Vanillosmopsis arborea* Baker essential oil (EOVA) and to clarify the underlying mechanism, using the acute model of chemical (eye wiping) nociception in mice. EOVA (25 to 200 mg/kg; p.o. and topical) evidenced significant antinociception against chemogenic pain in the test model of formalin-induced neuroinflammatory pain. Local application of 5 M NaCl solution on the corneal surface of the eye produced a significant nociceptive behavior, characterized by eye wiping. The number of eye wipes was counted during the first 30 s. EOVA (25, 50, 100, and 200 mg/kg; p.o. and topical) significantly decreased the number of eye wipes. Naloxone, yohimbine, L-NAME, theophylline, glibenclamide, and ruthenium red had no effect on the antinociceptive effect of EOVA. However, ondansetron, p-chlorophenylalanine methyl ester (PCPA), capsazepine, prazosin, and atropine prevented the antinociception induced by EOVA. These results indicate the topical antinociceptive effect of EOVA and showed that 5-HT, **α**1, TRPV1, and central muscarinic receptors might be involved in the antinociceptive effect of EOVA in the acute corneal model of pain in mice.

## 1. Introduction

The small size of the cornea and the extensive branching of the peripheral axons of corneal neurons make this structure the most densely innervated tissue of the body [1]. The majority of corneal sensory fibers are polymodal nociceptors, which are activated by noxious mechanical, thermal, and chemical stimuli [2].

Safe, long-lasting pain relief following corneal abrasions, corneal ulcers or ophthalmic surgery is difficult to achieve with current analgesics. The use of topical NSAIDs and acetominophen is constrained by their gradual onset and limited efficacy, and the adverse side effects of systemic opioids are well known [3].

*Vanillosmopsis arborea* Baker is native to the Araripe National Forest, in the Northeast of Brazil in the state of Ceará. There are few studies concerning the traditional use of this plant. However, biological and pharmacological studies have shown that its essential oil presents antimicrobial, antiinflammatory and gastroprotective activities [4].

Topically applied *Vanillosmopsis arborea *essential oil produced antinociception in acetic acid, cyclophosphamide, mustard oil, capsaicin or formalin-induced visceral pain [5]. The essential oil also suppressed the ear edema induced by Croton oil and phenol but no that one induced by histamine or capsaicin [6].

Ocular pain has not been adequately controlled in many patients and there is a lack of studies that examine the effect of *Vanillosmopsis arborea* on the perception of corneal pain. Therefore, the present study was designed to investigate the effects of acute topical administration of *Vanillosmopsis arborea *on acute corneal pain that was induced by hypertonic saline applied locally on the corneal surface in mice.

## 2. Material and Methods

### 2.1. Essential Oil

The essential oil from *Vanillosmopsis arborea *Baker bark (EOVA) was obtained from the Natural Products Research Laboratory of Regional University of Cariri. The composition (w/w) of EOVA revealed the presence of *α*-bisabolol to the extent of 70%. Other identified compounds were *α*-cadinol (8.4%), elemicin (6.21%), *β*-bisabolene (4.46%), *δ*-guaiene (2.31%), *β*-cubebene (1.76%), and estragole (1.08%).

### 2.2. Animals

Male Swiss albino mice (20–25 g) obtained from the Central Animal House of Regional University of Cariri were used. They were housed in environmentally controlled conditions (22°C, 12 h light-dark cycle), with free access to standard pellet diet (Purina, São Paulo, Brazil) and water. Animals were kept in cages with raised floors to prevent coprophagy. The experimental protocols were in accordance with the ethical guidelines of National Institute of Health, Bethesda, USA.

### 2.3. Formalin-Induced Paw Licking

The formalin-induced nociception was performed as described previously by Hunskaar and Hole [7]. Groups of mice (*n* = 8) were treated with vehicle (10 mL/kg, p.o.), EOVA (25, 50, 100, or 200 mg/kg p.o. or topical), or morphine (7.5 mg/kg, s.c.) 30 or 60 min before the administration of 20 *μ*L of 1% formalin (in 0.9% saline) into the plantar surface of the right hind paw. The duration of paw licking (s) as an index of painful response was determined at 0–5 min (early phase, neurogenic) and 20–25 min (late phase, inflammatory) after formalin injection.

### 2.4. Eye Wiping Test

Corneal nociception was induced by a local application of hypertonic saline to the corneal surface [8, 9]. One drop (40 *μ*L) of 5 M NaCl solution was applied locally on the corneal surface of mice using a fine dropper [10]. The number of eye wipes performed with the ipsilateral forepaw was counted for a period of 30 s. Topical (20 *μ*L/eye) or orally (10 mL/kg) EOVA (25, 50, 100 or 200 mg/kg; *n* = 8/group) or vehicle were given 1 h before the noxiou agent. Morphine 7 mg/kg (s.c.; *n* = 8) was used as positive control. A normal control group (*n* = 8) received one drop of 0.15 M NaCl (0.9%).

In order to verify the possible involvement of opioid, noradrenergic, nitrergic, 5-HT_3_, muscarinic, K_ATP_^+^, adenosinergic, and TRPV_1_ systems in the effect of EOVA, the animals (*n* = 8/group) were treated with the respective antagonist (Table 1), 30 min before the topical administration of EOVA (50 mg/kg). The doses of antagonists were chosen based on previous studies.

To assess the possible contribution of endogenous serotonin, animals (*n* = 8/group) were pretreated with *ρ*-chlorophenylalanine methyl esther (PCPA, 100 mg/kg, i.p., an inhibitor of serotonin synthesis) or with vehicle, once a day for 4 consecutive days. Then, 24 hours after the last PCPA or vehicle injection, animals received EOVA (50 mg/kg) and 1 h later they were tested in the eye wiping test.

### 2.5. Statistical Analysis

All data are presented as mean ± s.e.m. The data were evaluated by one-way analysis of variance with Student-Newman-Keuls post hoc test using the GraphPad Prism 4.0 statistical program. The level of significance was set at *P* < 0.05.

## 3. Results and Discussion

In formalin test, pretreatment with EOVA (oral and topical) caused significant diminutions of both first phase (neurogenic) and second phase (inflammatory) nociception responses (Tables 2(a) and 2(b)). Morphine (5 mg/kg), the reference standard, also significantly suppressed the formalin-response at both phases.

Topically administered EOVA (25, 50, 100, or 200 mg/kg) respectively (*P* < 0.001, *P* < 0.01, *P* < 0.01, and *P* < 0.0001, resp.) decreased the number of eye wipes induced by the local application of 5 M NaCl solution on the corneal surface (Table 3(a)). Oral treatment with EOVA also reduced the number of eye wipes (Table 3(b)).

EOVA was evaluated for topical antinociceptive activity in mice using experimental models of chemogenic nociception. EOVA (oral and topical) was effective in attenuating acute neurogenic and tonic inflammatory phases of the formalin response. The essential oil, given topically, elicited a dose-unrelated antinociceptive effect on the paw-licking response, just as observed in the eye wiping test.

The local application of a 5 M NaCl solution to the corneal surface produced corneal nociception. Previous work has shown that the application of hypertonic saline to the tongue and cornea transiently activates nociceptive neurons with wide dynamic range property in the trigeminal subnucleus caudalis [11]. Moreover, infusion of hypertonic saline into the masseter muscle produces hind paw shaking in addition to activating c-Fos positive neurons in the ipsilateral trigeminal subnucleus caudalis [12]. The results presented here are in agreement with previous findings [8, 9].

Study of trigeminal pain and analgesic effects on trigeminal acute pains such as headache, muscle spasms, dental problems, or postsurgery pain seems to be more problematic. The cornea is used for nociception studies in trigeminal system [13], since corneal nociceptive receptors have a large representation in the trigeminal ganglion through the ophthalmic branch of trigeminal nerve [14]. Thin myelinated fibres [15] as well as unmyelinated fibers in cornea respond to chemical, mechanical, and thermal noxious stimuli [16]. In rat, wiping the eye with forelimb is an obvious withdrawal response to corneal chemical stimuli. Some researchers have used eye wiping test for investigating the chemical pungency [17] or the presence of C-fiber activity [18, 19]. Eye wiping test is a phasic analgesic test and is sensitive to centrally acting analgesics. Hypertonic saline-induced corneal pain has been introduced as a model of acute pain for study of mechanisms of pain in the trigeminal system in rats [8].

The results of this investigation provide evidence that the essential oil from *V. arborea* bark is topically active in the attenuation of corneal pain induced by 5 M NaCl. Previous study showed the local antiinflammatory effect of the essential oil from *Vanillosmopsis arborea*. It was observed that EOVA effect can be related to release of leukotrienes, decrease the production of inflammatory eicosanoids and influence on the production of AA metabolites [6].

This antinociceptive effect may be related to the high *α*-bisabolol content in EOVA, since that *α*-bisabolol possess visceral antinociceptive activity [20] and it is able to reduce the neuronal excitability in a concentration-dependent manner [21].

In the present study, we attempted to characterize further some of the mechanisms through which EOVA exerts its antinociceptive action in chemical model of corneal pain in mice.

The antinociceptive effect induced by the EOVA (50 mg/kg) was significantly inhibited by ondansetron, PCPA, prazosin, atropine, and capsazepine (Tables 4, 5, 6, 7, and 8). On the other hand, the administration of glibenclamide, naloxone, ruthenium red, yohimbine, LNAME or theophylline did not prevent the EOVA-induced antinociception (Tables 7, 9, 10, 11, 12, and 13).

Serotoninergic neurons also play a crucial role in the control of pain [22] and the diversity of subtype receptors for serotonin makes this system able to exert either facilitatory or inhibitory function [23]. Spinal 5-HT_3_ receptors have been shown to mediate antinociception, possibly via GABA release [24, 25]. Concerning the mechanism through which EOVA exerts its antinociceptive action, the present study shows that the 5-HT_3_ receptor is likely involved. This conclusion derives from the fact that pretreatment of animals with the 5-HT_3_ antagonist, ondansetron, reversed the antinociception caused by EOVA.

In addition, the treatment of mice with tryptophan hydroxylase inhibitor (PCPA) at a dose known to decrease the cortical content of serotonin and to significantly reverse morphine antinociception [26, 27] attenuated the effect of EOVA, indicating the involvement of 5-HT in the antinociceptive effect of EOVA.

In the present study, the involvement of *α*_1_-receptors in the antinociceptive action of EOVA is suggested by the results showing that pretreatment of animals with prazosin (an *α*_1_-receptor antagonist) significantly blocked the antinociception caused by EOVA. Prazosin, a former selective *α*_1_-receptor antagonist, has high affinity for *α*_2B_-receptors [28]. It has been suggested that the effects of prazosin against spinal *α* agonists appear through *α*_2B_-receptors, although yohimbine interacts with *α*_2A_-receptor site as well as *α*_2B_-receptor site [29].

The roles of acetylcholine, cholinergic agonists, and cholinesterase inhibitors, collectively termed cholinomimetics, have been established in the modulation of pain and analgesia [30]. Here we show that the pretreatment of animals with atropine prevented the antinociceptive effect induced by EOVA. This result indicates that the muscarinic receptors are involved in EOVA-induced antinociceptive effect. It is already well established that the analgesic effect of systemic morphine is mediated by a descending cholinergic pathway [31] as well as spinal endogenous acetylcholine acting through muscarinic receptors [32–35].

Ruthenium red (a noncompetitive TRPV_1_ antagonist) did not affect the antinociceptive effect of EOVA. However, capsazepine (a competitive TRPV_1_ channel antagonist) inhibited this response, indicating that EOVA interacts directly with TRPV_1_ receptors may contribute to this antinociception.

In summary, we have demonstrated that topical EOVA reduces the nociceptive behavior in models of formalin-induced paw licking and eye wiping in mice. In the corneal pain model, the observed antinociception is possibly mediated by 5-HT, *α*_1_, muscarinic, and TRPV_1_ receptors.

## Figures and Tables

**Table 1:** Pharmacologic agents used for test in order to verify the possible action mechanism of EOVA antinociceptive effect.

Drug	Target	Dose (mg/kg)	Administration route
Naloxone	Opioid receptors	2 (11)	Intraperitoneal
Prazosin	*α*_1_-receptors	0.15 (12)	Intraperitoneal
Yohimbine	*α*_2_-receptors	2 (11)	Intraperitoneal
Theophylline	Adenosine receptors	5 (13)	Intraperitoneal
L-NAME	NO synthesis	2 (11)	Intraperitoneal
Ondansetron	5-HT_3_ receptors	0.5 (14)	Intraperitoneal
Atropine	Muscarinic receptors	0.1 (15)	Intraperitoneal
Glibenclamide	K_ATP_^+^ channels	2 (16)	Intraperitoneal
Ruthenium red	TRPV_1_ receptors	5 (13)	Intraperitoneal
(noncompetitive antagonist)		3 (11, 17, 18)	Subcutaneous
Capsazepine (competitive antagonist)		5 (16)	Intraperitoneal

**Table:** (a)

Group	Dose (mg/kg)	Paw licking time (s)	
		1st phase	2nd phase
Control	—	99.63 ± 5.95	41.75 ± 8.07
EOVA	25	106.8 ± 10.03	19.67 ± 8.83****
	50	81.83 ± 6.53****	4.66 ± 4.27****
	100	42.17 ± 8.63****	18.33 ± 6.83****
	200	26.75 ± 2.52****	24.83 ± 4.36****
Morphine	7.5	19.83 ± 4.33****	13.83 ± 4.56****

Data are expressed as mean ± s.e.m. ****P < 0.0001 compared to control group. ANOVA followed by Student-Newman-Keuls test.

**Table:** (b)

Group	Dose (mg/kg)	Paw licking time (s)	
		1st phase	2nd phase
Control	—	57.67 ± 1.89	96.17 ± 6.95
EOVA	25	32.00 ± 6.27****	49.33 ± 7.96****
	50	38.50 ± 4.23****	45.50 ± 9.78****
	100	34.83 ± 5.81****	36.50 ± 11.43****
	200	37.50 ± 4.08****	23.17 ± 10.51****
Morphine	7.5	19.83 ± 4.33****	13.83 ± 4.56****

Data are expressed as mean ± s.e.m. ****P < 0.0001 compared to control group. ANOVA followed by Student-Newman-Keuls test.

**Table:** (a)

Group	Dose (mg/kg)	Number of eye wipes (30 s)
Control	—	20.33 ± 0.66
EOVA	25	15.33 ± 0.91***
	50	16.67 ± 0.84**
	100	16.67 ± 0.66**
	200	14.33 ± 0.61****
Morphine	7.5	12.17 ± 0.83****

Data are expressed as mean ± s.e.m. **P < 0.01, ***P < 0.001, and

****P < 0.0001 compared to control group. ANOVA followed by Student-Newman-Keuls test.

**Table:** (b)

Group	Dose (mg/kg)	Number of eye wipes (30 s)
Control	—	22.13 ± 1.31
EOVA	25	16.50 ± 1.26**
	50	14.75 ± 0.70***
	100	15.25 ± 1.09***
	200	12.63 ± 0.73***
Morphine	7.5	11.88 ± 0.83***

Data are expressed as mean ± s.e.m. **P < 0.01, ***P < 0.001, and

****P < 0.0001 compared to control group. ANOVA followed by Student-Newman-Keuls test.

**Table 4:** Effect of pretreatment with ondansetron on the EOVA-induced corneal antinociception in mice.

Group	Dose (mg/kg)	Number of eye wipes (30 s)
Control	—	12.50 ± 0.67
OEVA	50	8.33 ± 1.05***
Ondansetron	0.5	5.00 ± 0.51****
+OEVA	50	12.50 ± 1.91

Data are expressed as mean ± s.e.m. ***P < 0.001 and ****P < 0.0001 compared to control group. ANOVA followed by Student-Newman-Keuls test.

**Table 5:** Effect of pretreatment with *ρ*-chlorophenylalanine methyl esther (PCPA) on the EOVA-induced corneal antinociception in mice.

Group	Dose (mg/kg)	Number of eye wipes (30 s)
Control	—	11.50 ± 0.84
OEVA	50	6.25 ± 0.16***
PCPA	100	9.12 ± 0.83
+OEVA	50	14.13 ± 0.35***

Data are expressed as mean ± s.e.m. ***P < 0.001 compared to control group. ANOVA followed by Student-Newman-Keuls test.

**Table 6:** Effect of pretreatment with prazosin on the EOVA-induced corneal antinociception in mice.

Group	Dose (mg/kg)	Number of eye wipes (30 s)
Control	—	14.17 ± 0.70
EOVA	50	8.33 ± 1.05***
Prazosin	0.15	12.33 ± 0.80
+EOVA	50	13.33 ± 0.95

Data are expressed as mean ± s.e.m. ***P < 0.001 compared to control group. ANOVA followed by Student-Newman-Keuls test.

**Table 7:** Effect of pretreatment with atropine or glibenclamide on the EOVA-induced corneal antinociception in mice.

Group	Dose (mg/kg)	Number of eye wipes (30 s)
Control	—	17.00 ± 0.85
EOVA	50	11.83 ± 0.74***
Atropine	0.1	10.83 ± 0.87****
+EOVA	50	14.83 ± 0.542
Glibenclamide	2	6.66 ± 0.95****
+EOVA	50	11.50 ± 0.84***

Data are expressed as mean ± s.e.m. ***P < 0.001 and ****P < 0.0001 compared to control group. ANOVA followed by Student-Newman-Keuls test.

**Table 8:** Effect of pretreatment with capsazepine on the EOVA-induced corneal antinociception in mice.

Group	Dose (mg/kg)	Number of eye wipes (30 s)
Control	—	14.32 ± 0.71
EOVA	50	11.00 ± 0.57*
Capsazepine	5	10.67 ± 0.76*
+EOVA	50	12.83 ± 0.47

Data are expressed as mean ± s.e.m. *P < 0.05 compared to control group. ANOVA followed by Student-Newman-Keuls test.

**Table 9:** Effect of pretreatment with naloxone on the EOVA-induced corneal antinociception in mice.

Group	Dose (mg/kg)	Number of eye wipes (30 s)
Control	—	20.00 ± 0.68
EOVA	50	13.17 ± 0.79****
+Naloxone	2	11.50 ± 0.88****

Data are expressed as mean ± s.e.m. ****P < 0.0001 compared to control group. ANOVA followed by Student-Newman-Keuls test.

**Table 10:** Effect of pretreatment with ruthenium red on the EOVA-induced corneal antinociception in mice.

Group	Dose (mg/kg)	Number of eye wipes (30 s)
Control	—	12.50 ± 0.67
EOVA	50	8.33 ± 1.05*
Ruthenium red	3	5.83 ± 2.33**
+EOVA	50	5.16 ± 0.30**

Data are expressed as mean ± s.e.m. *P < 0.05 and **P < 0.01 compared to control group. ANOVA followed by Student-Newman-Keuls test.

**Table 11:** Effect of pretreatment with yohimbine on the EOVA-induced corneal antinociception in mice.

Group	Dose (mg/kg)	Number of eye wipes (30 s)
Control	—	16.14 ± 1.12
EOVA	50	12.29 ± 0.77**
Yohimbine	2	8.00 ± 1.66***
+EOVA	50	9.33 ± 0.51**

Data are expressed as mean ± s.e.m. **P < 0.01 and ***P < 0.001 compared to control group. ANOVA followed by Student-Newman-Keuls test.

**Table 12:** Effect of pretreatment with L-NAME on the EOVA-induced corneal antinociception in mice.

Group	Dose (mg/kg)	Number of eye wipes (30 s)
Control	—	19.00 ± 0.57
EOVA	50	11.50 ± 0.67****
L-NAME	10	17.50 ± 0.84
+EOVA	50	12.83 ± 0.65****

Data are expressed as mean ± s.e.m. ****P < 0.001 compared to control group. ANOVA followed by Student-Newman-Keuls test.

**Table 13:** Effect of pretreatment with theophylline on the EOVA-induced corneal antinociception in mice.

Group	Dose (mg/kg)	Number of eye wipes (30 s)
Control	—	17.00 ± 0.85
EOVA	50	10.33 ± 0.55****
Theophylline	5	7.33 ± 2.06****
+EOVA	50	9.16 ± 0.60****

Data are expressed as mean ± s.e.m. ****P < 0.001 compared to control group. ANOVA followed by Student-Newman-Keuls test.
